# Use of telehealth in the provision of after-hours palliative care services in rural and remote Australia: A scoping review protocol

**DOI:** 10.1371/journal.pone.0261962

**Published:** 2022-01-13

**Authors:** Pathmavathy Namasivayam, Dung T. Bui, Christine Low, Tony Barnett, Heather Bridgman, Pauline Marsh, Simone Lee

**Affiliations:** 1 School of Nursing, University of Tasmania, Hobart, Tasmania, Australia; 2 Centre for Rural Health, School of Health Sciences, University of Tasmania, Newnham, Tasmania, Australia; Universitat d’Alacante, SPAIN

## Abstract

**Introduction:**

After-hours services are essential in ensuring patients with life limiting illness and their caregivers are supported to enable continuity of care. Telehealth is a valuable approach to meeting after-hours support needs of people living with life-limiting illness, their families, and caregivers in rural and remote communities. It is important to explore the provision of after-hours palliative care services using telehealth to understand the reach of these services in rural and remote Australia. A preliminary search of databases failed to reveal any scoping or systematic reviews of telehealth in after-hours palliative care services in rural or remote Australia.

**Aim:**

To review and map the available evidence about the use of telehealth in providing after-hours palliative care services in Australian rural and remote communities.

**Methods:**

The proposed scoping review will be conducted using the Arksey and O’Malley methodological framework and in accordance with the Joanna Briggs Institute methodology for scoping reviews. The reporting of the scoping review will be guided by the Preferred Reporting Items for Systematic reviews and Meta-Analyses extension for Scoping Reviews (PRISMA-ScR). This review will consider research and evaluation of after-hours services using telehealth for palliative care stakeholders in rural and remote Australia. Peer reviewed studies and grey literature published in English from 2000 to May 2021 will be included. Scopus, Web of Science, CINAHL Complete, Embase via Ovid, PsycINFO via Ovid, Emcare via Ovid, Medline via Ovid, and grey literature will be searched for relevant articles. Titles and abstracts will be screened by two independent reviewers for assessment against the inclusion criteria. Data will be extracted and analysed by two reviewers using an adapted data extraction tool and thematic analysis techniques. Diagrams, tables, and summary narratives will be used to map, summarise and thematically group the characteristics of palliative care telehealth services in rural and remote Australia, including stakeholders’ perceptions and benefits and challenges of the services.

## Introduction

The World Health Organization defines palliative care as “an approach that improves the quality of life of patients (adults and children) and their families who are facing problems associated with life-threatening illness. It prevents and relieves suffering through the early identification, correct assessment and treatment of pain and other problems, whether physical, psychosocial or spiritual” [1]. Every year, an estimated 40 million people need palliative care worldwide, but it is received by only 14% [1]. Palliative care can be provided in various settings, such as the person’s home, residential care facilities, hospitals and in-patient specialist palliative care units or hospice care facilities. The setting is dependent on the person’s situation, taking into consideration the severity of symptoms experienced; availability and extent of community and family support; emotional support received; mobility and access [2].

There is a growing demand for quality end of life care, and consequently health care services are finding themselves over-extended [3]. In Australia, 79,932 palliative care-related hospitalisations were reported between 2017–18 and there was a 16.9% increase in palliative care hospitalisations between 2013–14 and 2017–18 [4]. Australia has seen an increase in chronic and incurable illnesses due to population growth and growing ageing population. This has widened the patient group requiring palliative care. Furthermore, the growing ageing population in Australia has increased the need for palliative care. In 2017, there were 3.8 million Australians aged 65 and over (comprising 15% of the total population) increasing from 319,000 (5%) in 1927 and 1.3 million (9%) in 1977 [5, 6]. The number and proportion of older Australians is expected to continue to grow. By 2057, it is projected there will be 8.8 million older people in Australia (22% of the population); by 2097, 12.8 million people (25%) will be aged 65 and over [7]. As preparing for an ageing population and other unexpected stresses to our health care system, like COVID-19, Palliative Care Australia called for a serious look at reforming the health care system and innovative ways to provide palliative to meet the communities’ needs [8].

Recent reports highlight an inconsistent provision of palliative and end-of-life care across Australia, particularly in rural and remote communities [9, 10]. People living in rural and remote communities often face poorer health outcomes compared to people living in metropolitan areas in Australia due to their geographical isolation. They have a shorter live span, higher level of disease burden and poorer access to health care services. Although people living in rural and remote communities have a higher burden of illness and disease risk factors, they are often not well served by palliative care services in general, and after-hours support in particular. After-hours palliative care services are services provided by generalist and specialist palliative care services after business hours for care which cannot wait until regular hours [11]. These are hours after 6pm to 8am on weekdays, before 8am and after 12pm on Saturdays and all-day Sundays and public holidays. For patients and their families being cared for at home, access to after-hours palliative care services can be problematic. Challenges in accessing palliative care services especially after-hours can contribute to health inequalities and can impact a patient’s quality of life [12, 13]. These challenges are further magnified for patients and families living in rural and remote communities when services are less accessible after-hours. This can be very distressing for patients and families especially when symptoms are not able to be managed, and appropriate care not provided in a timely manner.

A major challenge to accessing rural palliative care services relates to the geographical maldistribution of the workforce. According to Australian Institute of Health and Welfare, in 2017, 85.3% of specialist palliative medicine physicians worked in major cities, 7.3% worked in inner regional areas, and 6.1% worked in outer regional areas [4]. In nursing, 71.8% of palliative care nurses worked in major cities compared to 20.8% worked in inner regional areas [4]. According to the Royal Australian College of General Practitioners, a significant portion of rural general practitioners surveyed reported that their palliative care skills were not recognised at a local (72%) or state level (92%) [14].

In rural and remote communities, gaps in service provision means that families must often take on the caregiver’s role. In enabling continuity of patient care, families and carers require support from health care professionals [15]. After-hours palliative services are essential in ensuring patients and their families/carers are adequately supported. According to the Victoria Department of Health, the access to the after-hours palliative care services has been a requirement for all Victorian community palliative care services since 1997 [11]. It is important for after-hours palliative care services to be responsive to the needs of people living with a life-limiting illness, their families and carers [16]. Palliative Care Australia recommends that after-hours palliative care access be built into the resourcing of specialist palliative care services [16].

One of the approaches to providing after-hours access to palliative care services in rural and remote communities is through telehealth [16]. Telehealth refers to the use of telecommunication technologies for the purpose of delivering health care services and related processes (such as health education) over the distance [17, 18]. Recent systematic reviews revealed that telehealth has been used to connect patients and their caregivers to medical professionals remotely. Examples of telehealth services are telephone consultation, telephone advice and support lines, video consultation, remote patient monitoring [19–22]. Telehealth has shown to be extremely useful resource for palliative care providers working in rural communities. An Australia study by Baird-Bower et al. [23] found a telephone support service to be a valuable tool for everyone involved in the care of end-of-life patients. In the United States, telephone advice has been accepted as an important part of healthcare services, with necessary telephone protocols and guidelines in place. The regular telephone line was chosen in several telehealth interventions because the majority of the population continues to have landlines and the necessary infrastructure. Communication via Internet or cellular phones has substantial geographic variation in addition to higher implementation and operation costs.

Oliver et al. [24] reported that videophone technology was a feasible way to overcome geographic barriers to connect caregivers remotely to the hospice care team, which potentially improved symptom management for hospice patients. Demiris and colleagues [25] found that support via videophones was useful to significantly decrease anxiety scores and increase quality of life scores over time among patients and their family members. Demiris et al. [26] also found that the videophone was not inferior to face-to-face modes in delivering treatment therapies to patients with various conditions. This communication tool was also found easy to use for both older adults, hospice staff, and caregivers. However, some hospice staff face challenges in the installation of this technology in patients’ homes and in hospices due to a lack of technical understanding [25]. Additional challenges in using videophones include occasional technical problems, the reliance on the bandwidth of the phonelines and high system implementation and operation costs [26].

Internet-based devices that deliver telehealth in palliative care specifically have been tested globally. Chiang et al. [27] developed a telehealth device that could provide home-based monitoring and support for patients with chronic disease. Findings revealed that family caregivers who used telehealth devices had a significantly lower burden, higher stress mastery and better individual responsibility in caring for the patient as compared to the telephone only comparison group. Chih et al. [28] provided evidence on the effectiveness of using an online symptom reporting system in reducing caregivers’ emotional distress due to the timely response of caregiving needs in symptom management to clinicians. Notably, in an early palliative care intervention in the rural southern United States, Dionne-Odom et al. [29] reported that some patients and caregivers did not like the internet-based applications, and many caregivers lacked the skills in using internet-based technology.

In 2020, a new temporary telehealth services to the Medicare Benefits Schedule was added by the Australian Government to reduce the risk of patient–patient and patient–clinician transmission of COVID-19 [30]. Furthermore, the Australian Department of Health currently provides a range of subsidised telehealth specialist consultations for Australian living in rural and remote locations [31]. These consultations have attempted to bridge the distance between people living in rural and remote Australia with specialists located in major cities delivering real-time health consultations online. Providing after-hours palliative care services via telephone has been trialled and reported over the last two decades across different states resulting in reduced rates of ambulance calls, emergency department admissions [23, 32], and reducing the sense of isolation of rural families caring for a palliative patients at home [33]. However, the reported barriers to the application of after-hours services using telehealth in rural and remote communities are affordability, lack of visual feedback and difficulties in understanding different accents in health professionals [34].

As models of palliative care provision vary across urban-rural gradients in Australia [35], it is important to review and map the existing after-hours palliative care services using telehealth in rural and remote Australia to identify the challenges and benefits of providing this service to patients and their families in rural and remote communities.

A preliminary search of PROSPERO, MEDLINE, Cochrane Database of Systematic Reviews and Joanna Briggs Institute Database of Systematic Reviews and Implementation Reports conducted, did not identify any current or underway scoping reviews or systematic reviews on telehealth in after-hours palliative care services in rural and remote Australia. Thus, the scoping review design will be used to summarise, understand and disseminate research findings pertaining to telehealth use in after-hours palliative care services in rural and remote communities in Australia [36]. In addition, the scoping review will determine the feasibility, value and potential scope of undertaking a full systematic review [36].

### Review questions

What is known about the use of telehealth in providing after-hours palliative care services in rural and remote Australia?

Sub questions are as follows:

- What are the features of telehealth used in providing after-hours palliative care services in rural and remote Australia?
- What are the perceptions of stakeholders (services providers and receivers of care and carers) in using telehealth to provide after-hours palliative care services in rural and remote Australia?
- What are the benefits of after-hours palliative care services using telehealth in rural and remote Australia?
- What are the challenges of using telehealth in after-hours palliative care services in rural and remote Australia?

## Methods

The proposed scoping review will be conducted using the Arksey and O’Malley methodological framework [36] and in accordance with the Joanna Briggs Institute methodology for scoping reviews [37]. The scoping review will be guided by the Preferred Reporting Items for Systematic reviews and Meta-Analyses extension for Scoping Reviews (PRISMA-ScR) [38]. The PRISMA-ScR checklist will be used to increase methodology transparency (S1 Table) [38]. The complete pre-registration of our study can be found at https://osf.io/4qs5j.

### Inclusion criteria

#### Participants

This scoping review will consider studies and grey literature that refer to the stakeholders of after-hours palliative care services using telehealth, including service providers and people receiving the services (service receivers, patients, and carers). Providers are people who deliver the services, and those who coordinate after-hours palliative care to patients. Providers include primary health care providers, community service providers, palliative care specialists, health managers. Receivers are patients of all ages, any gender, and any culture who have used the services and their caregivers/families, living in regional, rural, and remote communities.

#### Concept

This review will consider studies that explore the provision of after-hours palliative care services using telehealth. These services must use telehealth in delivering service provision that aims to provide accessible and effective care for people whose health condition cannot wait for treatment until regular face-to-face care services are next available. Although Palliative Care Australia refers to after-hours as outside 8 am to 6 pm weekdays, outside 8 am to 12 noon on Saturdays and all day on Sundays and public holidays [16] this review, will include studies that use the term after-hours. The selected studies must include at least one variable related to the stakeholders’ perception, benefits, or challenges of the services. Palliative care services that are not provided after-hours or do not use telehealth will be excluded from this review.

#### Context

This review will consider studies on the after-hours palliative care services using telehealth delivered in rural and remote Australian communities, which are all areas outside Australia’s major cities. The Modified Monash Model (MMM) will be used to define the locations of regional, rural and remote areas, which classifies areas as MM1-7 [39]. Classification according to the MMM is based on population density and proximity to services. Regional centres (MM 2), including inner and outer regional areas, are in or within a 20km drive of a town with over 50,000 residents. Large rural towns (MM 3), including inner and outer regional areas, are not MM 2 and are in or within a 15km drive of a town between 15000 to 50000 residents. Medium rural towns (MM 4), including inner and outer regional areas, are not MM 2 or MM 3 and are in, or within a 10km drive of a town with between 5000 to 15000 residents. Small rural towns (MM 5) are all remaining inner and outer regional areas. Remote communities (MM 6) are remote mainland areas and remote islands less than 5km offshore. Very remote communities (MM 7) are very remote areas. This review will include studies in locations which are in the areas within MM2 to MM7.

#### Types of sources

Data sources will include quantitative, qualitative, and mixed methods primary research studies published in journals and conference proceedings. In addition, research reports and government reports published in government and professional association websites relating to the topic will be included. Review papers will be excluded from the scoping review. Books, editorials, letters, and commentaries will also be excluded. Articles published in the last 20 years from January 2000 to May 2021 in English will be included, due to the increasing trend of government initiatives and funding in supporting telehealth research in Australia from the year 2000.

### Search strategy

The initial limited search of MEDLINE, CINAHL, and Scopus was undertaken to identify articles on the topic. The text words contained in the titles and abstracts of relevant articles, and the index terms used to describe the articles were used to develop a full search strategy for Medline (Complete), CINAHL (Complete), Scopus, Web of Science, Embase (Ovid), PsycINFO (Ovid), and Emcare (Ovid). Table 1 presents a full search strategy for MEDLINE (Complete) that was conducted in May 2021.

**Table 1:** Search strategy for MEDLINE complete.

Search	Query	Records retrieved
#1	TX telehealth OR telemedicine OR telemonitor* OR telepractice OR telenursing OR telecare OR mobile health OR mHealth OR eHealth OR e-Health OR telecare OR virtual care OR teletherapy OR telepractice OR teleconsult* OR remote consult* OR distance counsel* OR telecommunicat* OR telecommut* OR teleassist* OR teleconferenc* OR videoconferenc* OR videotelephon* OR telephone triage OR telephone assessment OR telephone advice OR Internet	460,868
#2	TX palliative* OR end of life* OR terminal* OR hospice* OR dying OR death	3,167,173
#3	TX after hours OR after-hours OR out of hours OR on call	987,988
#4	TX rural OR remote OR suburban OR regional OR bush OR outback OR outskirts	1,631,000
#5	TX Australia*	1,247,872
#6	#1 AND #2 AND #3 AND #4 AND #5	6,556
#7	**Limiters** - Date of Publication: 20000101–20210531	6,441
#8	**Narrow by Language: **- English	6,402
#9	**Narrow by Subject Geographic: **- Australia	240

The search strategy, including all identified keywords and index terms, will be adapted for each included information source. A professional librarian used the Peer Review of Electronic Search Strategies (PRESS) 2015 evidence-based guideline and checklist to review the search strategy [40].

A targeted search of grey literature will also be conducted in local organisations’ websites such as Care Search and Palliative Care Australia. Search terms will be applied on the website “search column” of the website. Publications on these websites will be screened for their relevance to identify potentially relevant literature on after-hours palliative care services using telehealth in rural and remote Australia.

Two reviewers will undertake the search in collaboration. In addition, reference lists of the selected articles will be scanned by the reviewers for additional articles.

### Study selection

**Fig 1:**
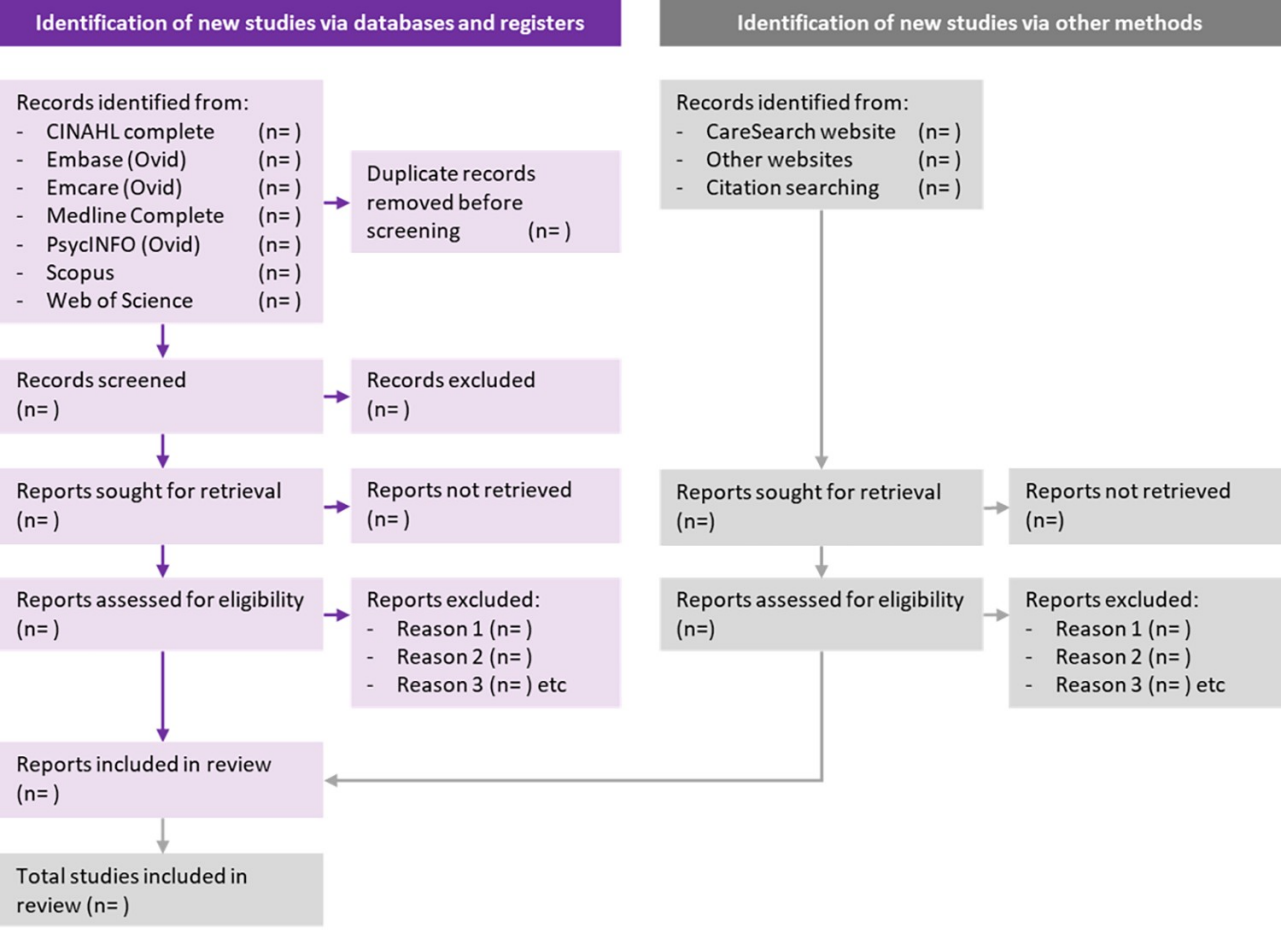
PRISMA 2020 flow diagram of the search strategy.

Following the search, all identified records will be collated and uploaded into EndNote 20 and all duplicates will be removed. Titles and abstracts will then be screened independently by two reviewers for assessment against the inclusion criteria for the review. Potentially relevant papers will be retrieved in full, and their citation details imported into Microsoft Excel. The full text of selected citations will be assessed independently in detail against the inclusion criteria by two reviewers. The outcomes of the screening process of each reviewer will be compared to each other on completion of each screening stage. Reasons for exclusion of full text papers that do not meet the inclusion criteria will be recorded and reported in the scoping review. Any disagreements that arise between the reviewers at each stage of the selection process will be resolved through discussion with a third reviewer. The study selection process and reasons for exclusion of publications will be presented in the updated PRISMA 2020 (Preferred Reporting Items for Systematic Reviews and Meta-analyses) flow diagram (Fig 1) [41].

### Data extraction

Data will be extracted from papers included in the scoping review by two authors using an adapted data extraction tool from Arksey and O’Malley [36]. The extracted data will include specific details about the population and sample, setting, methodology, outcome measurements and results which include (1) the features of telehealth; 2) stakeholders’ perceptions; (3) benefits of the services using telehealth; and (4) challenges of the services using telehealth. The data extracting tool will initially be tested with the first two articles and will be modified as appropriate during the process of extracting data from each included paper. Any potential disagreements that arise during data extraction between the reviewers will be resolved through discussion with a third reviewer. Authors of papers will be contacted to request missing or additional data, where required.

### Data presentation

The extracted data will be presented in diagrammatic and tabular form that aligns with the objective of this review. Diagrams, charts, or tables will be developed to fully illustrate the characteristics of telehealth used in providing the services, stakeholders’ perceptions, and the service benefits and challenges. Descriptive themes will be developed using the first two steps of thematic synthesis [42]. A narrative report will be presented to summarise the extracted data. The themes pertinent to the research questions will be described in the narrative; telehealth, service and user related features; service and user benefits; service and user challenges. These categories will organise the results in relation to the review questions in more detail and in the context of the overall study purpose. Table 2 shows the summary table of the included articles.

**Table 2:** Summary table.

Author(s) (Year)	Aims	Design, sample, & locations	Services & duration	General findings
…	…	…	…	…

## Discussion

The growing ageing population and other factors have put an increasing demand on health care services. Furthermore, people living in remote and isolated communities have high levels of illness and chronic conditions. However, services and specialist palliative care networks often have limited capacity to support patients and their families [4, 43]. Across Australia, there are 242 specialists palliative care services, and they were mostly located in major cities and large urban areas [44]. The limited access to services and resources makes the delivery of quality palliative care services challenging [45]. People living in rural and remote communities in Australia often experience poor access to palliative especially during after-hours. Poor access to palliative care services especially after-hours can be very distressing for both patients and families especially. This can lead to patients’ symptoms not being managed in a responsive way that meets patients’ and families’ needs. By enabling communities in rural and remote communities in Australia to have access to immediate after-hours palliative care services, telehealth can be useful in servicing these communities and overcoming issues of access and availability [16].

Telehealth has been used to provide after-hours palliative care service to communities in the rural and remote areas. It can overcome geographical challenges by delivering healthcare outside of traditional health care facilities [26]. It is cited as an extremely useful resource for palliative care givers and community nurses working in rural communities. In Australia, providing after-hours palliative cares via telephone has been trialled and reported during the last two decades across states, for examples, New South Wales [32, 33, 46], Victoria [47, 48], Queensland [49], and Tasmania [23]. However, the use of telephone in providing services has its challenges. Being a traditional audio-only communication tool, it prevents nonverbal interaction, can cause communication difficulties, and cannot provide visual feedbacks [34]. Furthermore, the 2012 after-hours palliative care framework published by the Victoria State Government pointed out that clients and carers accessing the telephone support services raised concerns of its use [11].

### Strengths and limitations of study

The review will be limited to rural and remote communities as it aims to review and map the available evidence [36] about the use of telehealth in providing after-hours palliative care services in Australian rural and remote communities. As the models of after-hours palliative care services using telehealth in rural and remote Australia vary [50], the findings of this review will provide a mechanism to summarising and disseminating the research findings for service users, service providers and researchers. Another limitation of this review is that we may not include all the terms relating to the search terms that we are not aware of. As this is a scoping review, critical analysis and synthesis of data will not be undertaken, therefore recommendations for policy makers cannot be provided.

## Supporting information

S1 Table PRISMA-ScR checklist. (ZIP)

## References

[1] World Health Organization. Palliative care [Internet]. [updated 2020 Aug 5; cited 2021 Apr 30]. Available from: https://www.who.int/news-room/fact-sheets/detail/palliative-care.

[2] Palliative Care Tasmania. Palliative care [Internet]. Griffith ACT [cited 2020 Jul 1]. Available from: https://www.pallcaretas.org.au/palliative-care/.

[3] Grindrod A, Rumbold B. Healthy End of Life Project (HELP): a progress report on implementing community guidance on public health palliative care initiatives in Australia. Ann Palliat Med. 2018:S73–S83. doi: 10.21037/apm.2018.04.01 29764174

[4] Australian Institute of Health and Welfare. Palliative care services in Australia [Internet]. Canberra ACT [updated 2020 Jun 25; cited 2020 Jul 1]. Available from: https://www.aihw.gov.au/reports/palliative-care-services/palliative-care-services-in-australia/contents/overview.

[5] Australian Bureau of Statistics. Australian historical population statistics. Canberra: ABS, 2014 3105.0.65.001.

[6] Australian Bureau of Statistics. Australian Demographic Statistics. Canberra: ABS, 2017 3101.0.

[7] Australian Institute of Health and Welfare. Older Australia at a glance. Canberra ACT: AIHW, 2018 AGE 87.

[8] Palliative Care Australia. Calls for palliative care reform in post COVID-19 world. Griffith ACT: PCA, 2020.

[9] Crawford M. General’s report–Performance audit: Planning and evaluating palliative care services in NSW. Sydney NSW: Audit Office of NSW, 2017.

[10] Wenham S, Cumming M, Saurman E. Improving palliative and end-of-life care for rural and remote Australians. Public Health Res Pract. 2020;30(1):e3012001. doi: 10.17061/phrp3012001 32152613

[11] Palliative Care team. After-hours palliative care framework. Melbourne VIC: Integrated Care Branch, Wellbeing Integrated Care and Ageing Division, the Department of Health, State of Victoria, 2012.

[12] Australian Institute of Health and Welfare. Rural & remote health [Internet]. Canberra ACT [updated 2019 Oct 22; cited 2020 Jul 1]. Available from: https://www.aihw.gov.au/reports/rural-remote-australians/rural-remote-health/contents/summary.

[13] Bradford N, Caffery L, Smith A. Telehealth services in rural and remote Australia: A systematic review of models of care and factors influencing success and sustainability. Rural Remote Health. 2016;16(4):3808. Epub 2016 Oct 17. PubMed Central PMCID: PMC27744708. 27744708

[14] Royal Australian Coffege of General Practitioners. GP-led palliative care in rural Australia. East Melbourne VIC: 2016.

[15] CareSearch. Rural and remote (Specific populations) [Internet]. [updated 2020 Jun 2; cited 2020 Jul 1]. Available from: https://www.caresearch.com.au/caresearch/tabid/181/Default.aspx.

[16] Palliative Care Australia. Palliative care service development guidelines. Griffith ACT: PCA, 2018.

[17] WHO. Telehealth: World Health Organization; 2019 [cited 2019 May 20]. Available from: https://www.who.int/sustainable-development/health-sector/strategies/telehealth/en/.

[18] The Australian Department of Health. Telehealth 2015 [cited 2019 20 Dec]. Available from: https://www1.health.gov.au/internet/main/publishing.nsf/Content/e-health-telehealth.

[19] Chi NC, Demiris G. A systematic review of telehealth tools and interventions to support family caregivers. J Telemed Telecare. 2015;21(1):37–44. Epub 2014 Dec 4. doi: 10.1177/1357633X14562734 . 25475220 PMC4486048

[20] Zheng Y, Head BA, Schapmire TJ. A systematic review of telehealth in palliative care: Caregiver outcomes. Telemed J E Health. 2016;22(4):288–94. doi: 10.1089/tmj.2015.0090 26360181

[21] Disalvo D, Agar M, Caplan G, Murtagh FE, Luckett T, Heneka N, et al. Virtual models of care for people with palliative care needs living in their own home: A systematic meta-review and narrative synthesis. Journal of Palliative Medicine. 2021:2692163211024451. doi: 10.1177/02692163211024451 . 34169759

[22] Johnston BM, McCauley R, McQuillan R, Rabbitte M, Honohan C, Mockler D, et al. Effectiveness and cost-effectiveness of out-of-hours palliative care: a systematic review. HRB Open Res. 2020;3:9. Epub 2021/02/16. doi: 10.12688/hrbopenres.13006.1 ; PubMed Central PMCID: PMC7845148. 33585789 PMC7845148

[23] Baird-Bower D, Roach J, Andrews M, Onslow F, Curnin E. Help is just a phone call away: After-hours support for palliative care patients wishing to die at home. International journal of palliative nursing. 2016;22(6):286–91. doi: 10.12968/ijpn.2016.22.6.286 . 27349847

[24] Oliver DP, Demiris G, Wittenberg-Lyles E, Porock D, Collier J, Arthur A. Caregiver participation in hospice interdisciplinary team meetings via videophone technology: A pilot study to improve pain management. Am J Hosp Palliat Care. 2010;27(7):465–73. doi: 10.1177/1049909110362402 . 20299692 PMC2890035

[25] Demiris G, Oliver DP, Courtney KL, Day M. Telehospice tools for caregivers—A pilot study. Clin Gerontologist. 2007;31(1):43–57. doi: 10.1300/J018v31n01_04

[26] Demiris G, Parker Oliver D, Wittenberg-Lyles E, Washington K, Doorenbos A, Rue T, et al. A non-inferiority trial of a problem-solving intervention for hospice caregivers: In person versus videophone. Journal of Palliative Medicine. 2012;15(6):653–60. doi: 10.1089/jpm.2011.0488 . 22536989 PMC3362957

[27] Chiang LC, Chen WC, Dai YT, Ho YL. The effectiveness of telehealth care on caregiver burden, mastery of stress, and family function among family caregivers of heart failure patients: A quasi-experimental study. Int J Nurs Stud. 2012;49(10):1230–42. doi: 10.1016/j.ijnurstu.2012.04.013 . 22633448

[28] Chih MY, DuBenske LL, Hawkins RP, Brown RL, Dinauer SK, Cleary JF, et al. Communicating advanced cancer patients’ symptoms via the Internet: A pooled analysis of two randomized trials examining caregiver preparedness, physical burden, and negative mood. Journal of Palliative Medicine. 2013;27(6):533–43. doi: 10.1177/0269216312457213 . 22988042 PMC3819140

[29] Dionne-Odom JN, Taylor R, Rocque G, Chambless C, Ramsey T, Azuero A, et al. Adapting an early palliative care intervention to family caregivers of persons with advanced cancer in the rural deep south: A qualitative formative evaluation. J Pain Symptom Manage. 2018;55(6):1519–30. doi: 10.1016/j.jpainsymman.2018.02.009 . 29474939 PMC5951755

[30] Snoswell CL, Caffery LJ, Haydon HM, Thomas EE, Smith AC. Telehealth uptake in general practice as a result of the coronavirus (COVID-19) pandemic. Aust Health Rev. 2020;44(5):737–40. doi: 10.1071/AH20183 32853536

[31] Australian Department of Health. Telehealth pilots programme [Internet]. [updated 2014 Feb 25; cited 2020 Jul 1]. Available from: https://www1.health.gov.au/internet/main/publishing.nsf/Content/ehealth-nbntelehealth-pilots.

[32] Phillips JL, Davidson PM, Newton PJ, Digiacomo M. Supporting patients and their caregivers after-hours at the end of life: the role of telephone support. J Pain Symptom Manage. 2008;36(1):11–21. doi: 10.1016/j.jpainsymman.2007.08.017 . 18411012

[33] Wilkes L, Mohan S, White K, Smith H. Evaluation of an after hours telephone support service for rural palliative care patients and their families: A pilot study. Aust J Rural Health. 2004;12(3):95–8. doi: 10.1111/j.1440-1854.2004.00568.x . 15200518

[34] Wadhwa A, Lingard L. A qualitative study examining tensions in interdoctor telephone consultations. Med Educ. 2006;40(8):759–67. doi: 10.1111/j.1365-2929.2006.02534.x . 16869921

[35] National Rural Health Alliance. Palliative care in rural and remote areas—Fact sheet 34. Deakin West, ACT: 2012.

[36] Arksey H, O’Malley L. Scoping studies: Towards a methodological framework. Int J Soc Res. 2005;8(1):19–32. doi: 10.1080/1364557032000119616

[37] Peters MD, Godfrey C, McInerney P, Munn Z, Tricco AC, Khalil H. Chapter 11: Scoping Reviews (2020 version). In: Aromataris E, Munn Z, editors. JBI Manual for Evidence Synthesis: JBI; 2020.

[38] Tricco AC, Lillie E, Zarin W, O’Brien KK, Colquhoun H, Levac D, et al. PRISMA Extension for Scoping Reviews (PRISMA-ScR): Checklist and Explanation. Annals of internal medicine. 2018;169(7):467–73. doi: 10.7326/M18-0850 30178033

[39] Australian Department of Health. Modified Monash model [Internet]. [updated 2020 Jan 9; cited 2020 Jul 1]. Available from: https://www.health.gov.au/health-workforce/health-workforce-classifications/modified-monash-model.

[40] McGowan J, Sampson M, Salzwedel DM, Cogo E, Foerster V, Lefebvre C. PRESS Peer Review of Electronic Search Strategies: 2015 Guideline Statement. J Clin Epidemiol. 2016;75:40–6. doi: 10.1016/j.jclinepi.2016.01.021 27005575

[41] Page MJ, McKenzie JE, Bossuyt PM, Boutron I, Hoffmann TC, Mulrow CD, et al. The PRISMA 2020 statement: an updated guideline for reporting systematic reviews. BMJ. 2021;372:n71. doi: 10.1136/bmj.n71 33782057 PMC8005924

[42] Thomas J, Harden A. Methods for the thematic synthesis of qualitative research in systematic reviews. BMC Med Res Methodol. 2008;8(1):45. doi: 10.1186/1471-2288-8-45 18616818 PMC2478656

[43] Australian Department of Health and Human Services. Tasmanian model of services delivery for palliative care [Internet]. [updated 2007 July; cited 2019 17 March]. Available from: https://www.dhhs.tas.gov.au/palliativecare/better_access_to_palliative_care_project_outcomes_2012_-_2017/project_activities/tasmanian_model_of_service_delivery_for_palliative_care.

[44] Palliative Care Australia. Palliative Care Australia’s submission to the preliminary report for consultation: urgent after-hours primary care services funded through the MBS. Griffith ACT: PCA, 2017.

[45] CRANA plus. CRANA plus position paper: Palliative care. Cairns QLD: 2015.

[46] Cromwell D, Senior K, Owen A, Gordon R, Eagar K. Can the National Palliative Care Strategy be translated into a model of care that works for rural Australia? An answer from the Griffith Area Palliative Care Service experience. Wollongong: Centre for Health Service Development, University of Wollongong, 2003.

[47] Ciechomski L, Tan H, O’Connor M, Miles G, Schattner P, Klein B. After hours palliative care provision in rural and urban Victoria. Asia Pac J Health Manag. 2009;4(1):41–6.

[48] Victoria Department of Health & Human Services. After hours palliative care pilot projects: Evaluation report. Victoria, Australia: Services DoHH; 2013.

[49] Bradford N, Irving H, Smith AC, Pedersen LA, Herbert A. Palliative care afterhours: A review of a phone support service. J Pediatr Oncol Nurs. 2012;29(3):141–50. Epub 2012/06/01. doi: 10.1177/1043454212446023 . 22647726

[50] Armstrong K, Amoyal G, Jacups DS, Verhoeven A. Review of after-hours service models: Learnings for regional, rural and remote communities. Health Future Australia, 2016.

