# Supplementary figures and images for: Use of telehealth in the provision of after-hours palliative care services in rural and remote Australia: A scoping review protocol
Source: PLoS One. 2022 Jan 13;17(1):e0261962. doi: 10.1371/journal.pone.0261962 (PMC8758106; doi:10.1371/journal.pone.0261962)

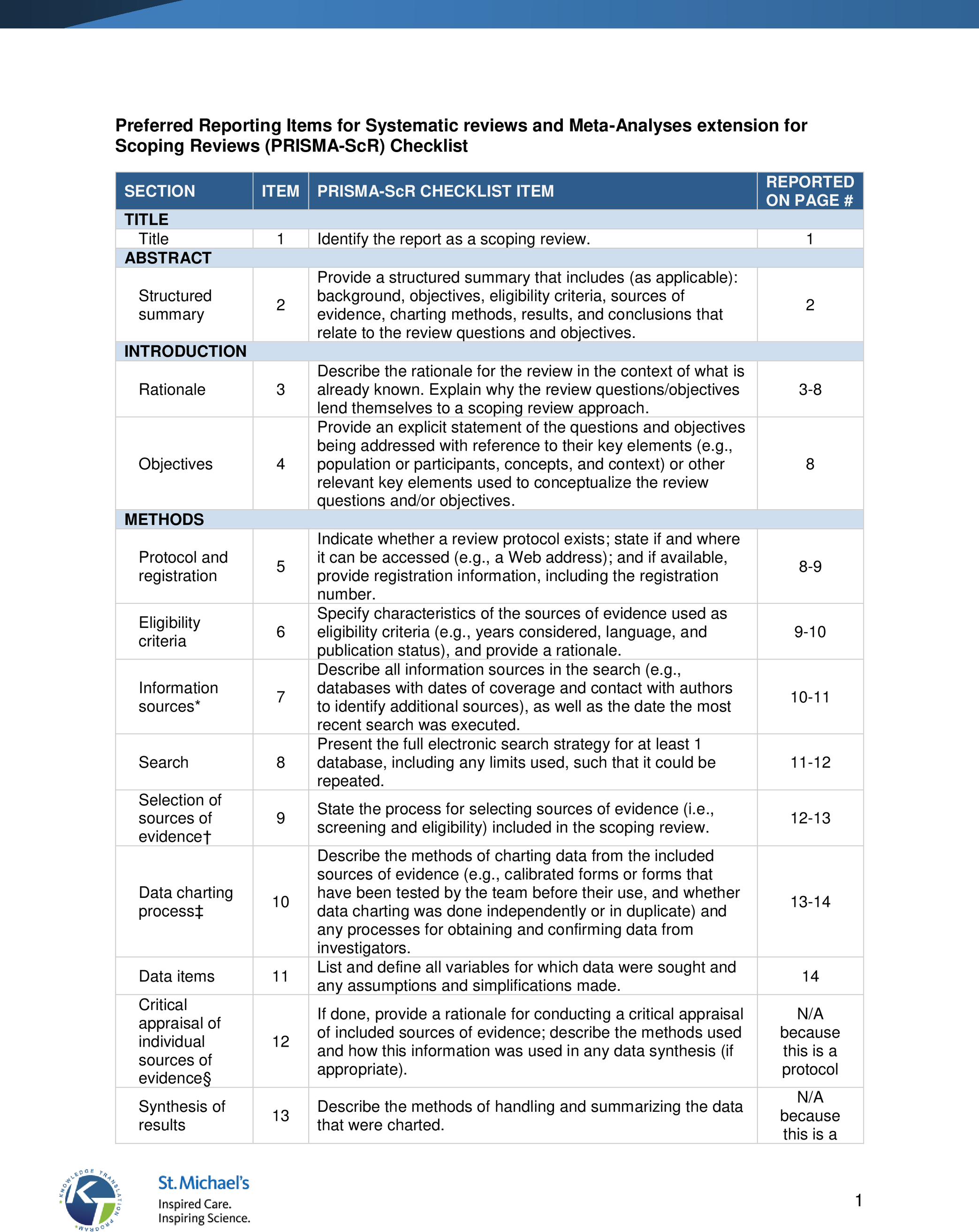

Supplement: S1 Table — (ZIP) [file pone.0261962.s001.zip › S1A_Table.tif]

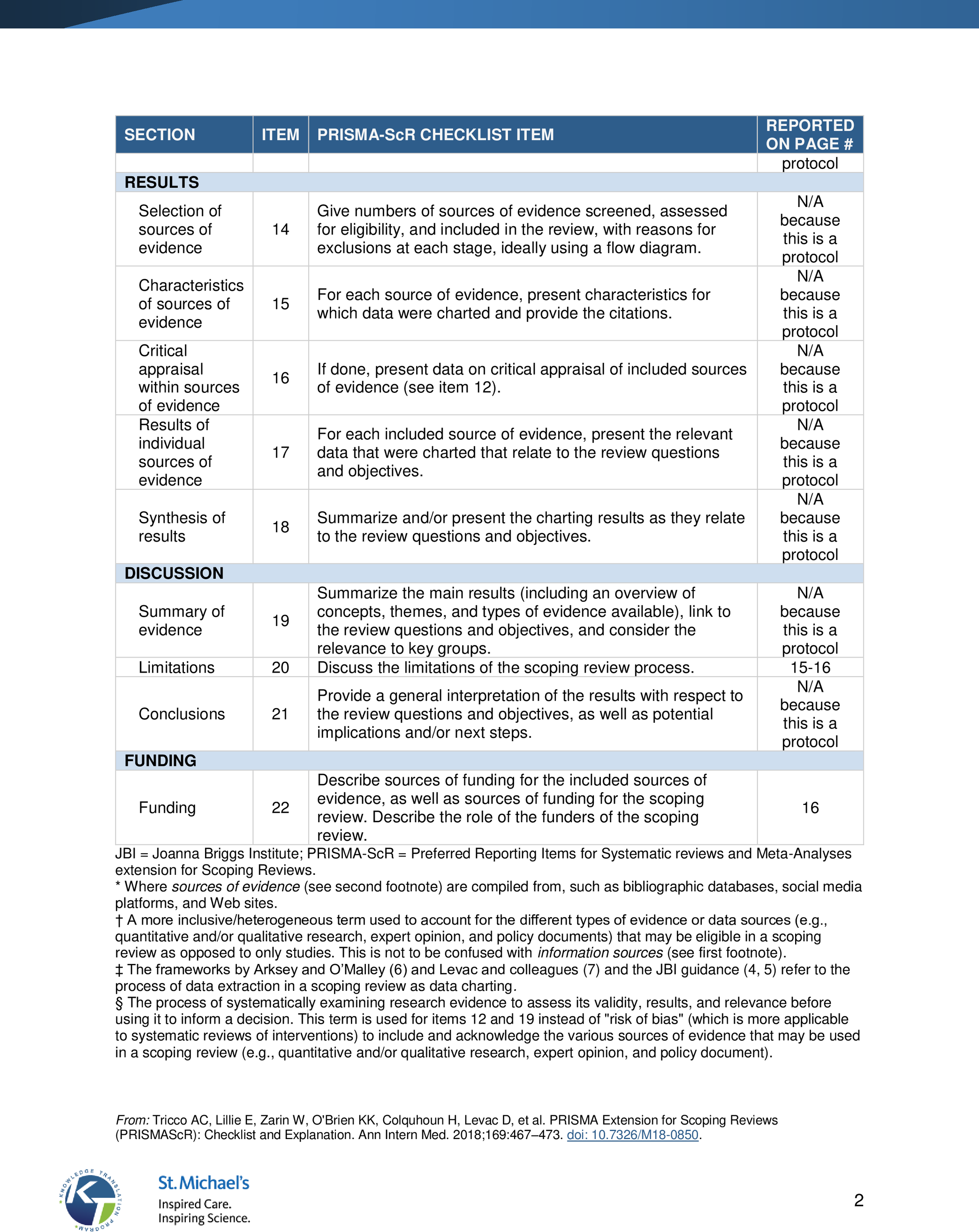

Supplement: S1 Table — (ZIP) [file pone.0261962.s001.zip › S1B_Table.tif]
